# Fetal in-utero management of myelomeningocele: a mini-review on history, challenges, management gap, and recommendations

**DOI:** 10.1097/MS9.0000000000002061

**Published:** 2024-04-19

**Authors:** Areeba Fareed, Solay Farhat, Abed AlRazzak Kerhani, Anood Choudhary, Syeda Sadia Masood Raza

**Affiliations:** a Karachi Medical and Dental College, Karachi, Pakistan; b Faculty of Medical Sciences, Lebanese University; c Faculty of Medicine, University of Balamand, Beirut, Lebanon

Neural tube defects (NTDs), while possibly preventable, are amongst the most common congenital anomalies, accounting for a significant portion of prenatal mortality and morbidity. Indeed, they represent a complex array of congenital anomalies stemming from the intricate failure of neural tube closure during embryonic development, thereby exposing critical neurodevelopmental structures to amniotic fluid^1^. Undoubtedly, the etiology of NTDs is multifaceted, encompassing both primary and secondary neurulation deficiencies. Primary neurulation involves the intricate folding of the dorsal neural tube, laying the groundwork for the genesis of the brain and spinal cord, while secondary neurulation entails neural tube canalization and subsequent maturation of the dorsal spinal cord^2^. Any disruption in these processes invariably precipitates the emergence of NTDs, manifesting in either open or closed forms. The dichotomy between open and closed NTDs accentuates the heterogeneity within this class of developmental disorders. Open NTDs, comprising craniorachischisis, myelomeningocele (MMC), lipomyelomeningocele, and related conditions, lack epithelial coverings over neural structures. Conversely, closed NTDs, which encompass entities like encephalocele, spina bifida occulta, split cord malformation, and various other presentations, demonstrate an epithelial covering over the affected neural tube structures^3^. Apart from their intricate embryological nature, NTDs represent a considerable global public health challenge, significantly contributing to fetal mortality and neonatal disabilities^4^. The documented incidence of NTDs ranges from 1 in 800 to 1 in 1000 live births, with some geographic and demographic variations. Additionally, NTDs mortality rates can reach up to 65–70% in the initial six months after birth if patients remain untreated^5^. Notably, MMC, a common subtype of NTDs, is one of the non-lethal conditions investigated for prenatal intervention^6^.

MMC is a significant neurological disorder originating from the insufficient sealing of the spinal neural tube in the initial phases of embryo development. This leads to the extrusion of neural tissue or meninges into a sac filled with fluid at the corresponding level of the spine^7^. Typically, the detection of MMC involves prenatal screening techniques such as standard ultrasound, fetal MRI, and fetal echocardiography. Specifically, a combination of maternal serum alpha-fetoprotein (AFP) screening and structural ultrasound examinations conducted between the 18th and 20th weeks of gestation can identify the anomaly in more than 80% of cases^8^. Furthermore, during routine comprehensive assessments of the brain, healthcare professionals may observe a distinct lemon-shaped deformity of the skull, which acts as a confirming sign for diagnosing MMC in the fetus^9^. Prenatal diagnosis allows for both proper neurosurgical treatment and perinatal planning depending on the abnormality’s presentation and progression^8^. As for the treatment, and in selected cases, prenatal surgery for open spina bifida is now being conducted, offering expectant mothers carrying a fetus with MMC an extra treatment option.

The inception of in-utero intervention, first conducted at the University of California, San Francisco (UCSF) in 1982 to mitigate a urinary tract obstruction, sparked immense excitement and marked a seminal moment in medical advancement. Yet, it also triggered serious debates about safety, effectiveness, ethical standards, as well as the importance of multidisciplinary collaboration^6^. As maternal safety improved, fetal intervention broadened to address an extensive array of conditions, ranging from minor fetal wounds to serious conditions like congenital diaphragmatic hernia, hence demonstrating the growing scope of fetal medicine^10^.

The first human fetal MMC in-utero repair was performed by Dr Joseph Bruner at Vanderbilt University in 1997, which became a basis for a randomized controlled trial that led to the multicenter Management of Myelomeningocele Study (MOMS). The MOMS trial evaluated both maternal and fetal morbidity and mortality perinatally, at 12 months, 30 months, and school-age (mean 7.8 years, range 5.9–10.3 years)^6,11–17^. In the MOMS trial, the mean duration of the fetal surgery was notable. The in-utero repair presented a decline in both the frequency as well as the severity of Arnold–Chiari II malformation among the fetuses. Regarding maternal health, even though future fertility was not affected, the fetal MMC repair appeared to be associated with a higher rate of preterm deliveries in subsequent pregnancies^6^.

Furthermore, the intervention may cause maternal complications such as pulmonary edema, chorioamniotic membrane separation, uterine dehiscence, and increased uterine contractility^11^. Since fetal surgery requires more aggressive manipulation of the membranes, PROM (Premature Rupture Of Membranes) is another possible complication^12^. To guarantee the safety of both, obstetrics and pediatric anesthesia teams must cooperate together to provide suitable local and general maternal anesthetic as well as fetal airway management. Moreover, to avoid uterine dehiscence, a strict plan remains in place, with the intervention conducted between 24 and 26 weeks of gestation and labor at 35 weeks, followed by a C-section^13^.

Some of the anesthetic struggles associated with open fetal procedures include profound uterine relaxation induction, maternal and fetal blood loss vigilance, fetal monitoring, fetal monitoring, and sometimes resuscitation^13^. Open repair also has a higher risk of maternal and/or fetal morbidity than less invasive methods. Open fetal surgery increases the risk of uterine dehiscence and premature labor, as well as preterm delivery and fetal demise^13^.

The management of fetal MMC is a crucial frontier in prenatal care, integrating advances in both diagnostics and therapeutic approaches. Prenatal diagnostic tools have improved to enable early diagnosis of MMC, allowing for appropriate decisions to mitigate related risks. The MOMS study found that prenatal closure reduced the need for shunting by 42% compared to postnatal therapy (*P*<0.05). Open prenatal MMC repair resulted in a 32% improvement in motor function (*P*<0.01) and a significant 58% reduction in hindbrain herniation (*P*<0.001). However, the scene of fetal MMC management is complex, with novel procedures such as percutaneous fetoscopic repair displaying both benefits and difficulties. For instance, percutaneous fetoscopic repair is associated with higher rates of premature membrane rupture (91% vs. 36% in open repair, *P*<0.01), preterm birth rates (96% vs. 81% in open repair, *P*=0.04), and repair site dehiscence or leakage (30% vs. 7% in open repair, *P*<0.01). Conversely, fetoscopic repair by maternal laparotomy reduces the risk of preterm delivery compared to percutaneous fetoscopic repair. Notably, open fetal MMC repair provides particular challenges, including a greater rate of uterine dehiscence (11% vs. 0% in fetoscopic repair, *P*<0.01). These statistical findings highlight the complexity of fetal MMC treatment, emphasizing the significance of precision and ongoing studies to enhance outcomes^14,15^.

As opposed to postnatal repair, fetal MMC repair offers potential long-term benefits, especially during young adulthood. The MOMS trial found that prenatally treated children had considerably better motor function (51.3% vs. 23.1% independent walking at 2 years) and required less shunting^16^. Studies showed better bladder/bowel control and independent toileting, implying improved everyday life and social engagement^17^. While cognitive outcomes require additional investigation, the potential socio-emotional advantages and similar cognitive development are promising^18^. Despite possible risks, the observed long-term advantages indicate a paradigm change in MMC treatment, necessitating further investigation to improve decision-making for afflicted families.

The guidelines for fetal MMC repair emphasize a multidisciplinary team approach, extensive prenatal evaluation using fetal MRI, precise surgical technique, and close postoperative monitoring^19^. Furthermore, it is suggested to provide all evidence-based treatment choices, such as antenatal or fetal closure, pregnancy termination, and adoption, with early consultation taking place between weeks 18 and 20 of gestation^20^. The surgical and obstetrical consequences should be carefully considered, and genetic testing by amniocentesis is recommended to guide decision-making about in-utero MMC closure. These recommendations emphasize the need for customized, educated decision-making, as well as continuous research and advances in fetal MMC management.

## Ethical approval

Not applicable.

## Consent

Not applicable.

## Sources of funding

The authors did not receive any financial support for this work. No funding has been received to conduct this study.

## Author contribution

All authors contributed equally.

## Conflicts of interest disclosure

The authors declare that there are no conflicts of interest.

## Research registration unique identifying number (UIN)

Not applicable.

## Guarantor

Solay Farhat, MD, Faculty of Medical Sciences, Lebanese University, Beirut, Lebanon; e-mail: solayfarhat@gmail.com, ORCID: 0000-0002-5108-7658.

## Data availability statement

The data that supports the findings of this study are available online^21^.

## Provenance and peer review

Not commissioned, externally peer-reviewed.
